# Influences of nutrition and metabolism on reproduction of the female ruminant

**DOI:** 10.21451/1984-3143-AR2018-0017

**Published:** 2018-08-03

**Authors:** Ana Meikle, Victoria de Brun, Mariana Carriquiry, Pablo Soca, Cecilia Sosa, María de Lourdes Adrien, Pablo Chilibroste, José Alfonso Abecia

**Affiliations:** 1 Facultad de Veterinaria, Udelar, Montevideo, Uruguay; 2 Facultad de Agronomía, Udelar, Montevideo, Uruguay; 3 Facultad de Veterinaria, Universidad de Zaragoza, Zaragoza, Spain

**Keywords:** metabolism, reproduction, ruminant.

## Abstract

Beef cows and ewes grazing native pastures are exposed to cycles of undernutrition that reflect the seasonal variations of biomass production. In grazing dairy cows, the physiological undernutrition during early lactation due to increased demands for lactation and low dry matter intake is exacerbated by the need to get sufficient intake from pasture and the extra grazing energy costs. Undernutrition has profound impacts on reproduction by affecting multiple reproductive processes at different levels of the reproductive axis. The objective of this paper is to review the influence of undernutrition on reproductive events of the adult female ruminant, with emphasis on both grassland and mixed rain-fed grazing farming systems. The comparative endocrinology and reproductive biology among ewes, beef and dairy cows may provide a comprehensive knowledge of the metabolic and reproductive adaptation to feed restriction. Understanding the critical underlying physiological mechanisms by which nutrition affects reproduction is the base of focus feeding strategy to improve the reproductive performance of the female ruminant.

## Introduction

The world’s population numbered nearly 7.6 billion in 2017, a large proportion of its increase has been in developing countries where livestock production is a major factor of agriculture growth (Food and Agriculture Organization - FAOSTATS, 2017). Despite the ability of herbivores to convert human-inedible fibrous biomass into human-edible food, the sustainability of this food production is under discussion (Gill *et al*., 2010). The innovations in livestock practices in the last decades resulted in increased animal production. However, the increase in the concentration of greenhouse gases and of minerals in surface and ground water in high-density livestock operations, illustrates that current livestock practices do not meet the definitions of environmental sustainability (Van Vuuren and Chilibroste, 2013). In this scenario, grasslands and mixed rain-fed systems (i.e., farming practices that rely on rainfall for water availability) constitute an alternative for sustainable livestock production. Moreover, maximizing the proportion of pastures in the diet is a pivotal factor for minimising production costs.

Grasslands (25% earth surface; FAOSTATS, 2017) are found primarily in marginal areas unfit for cropping. Native pastures are exposed to high variability in biomass production throughout the year due to normal seasonal variation and increasingly abnormal, extreme climate events. These seasonal variations, also influenced by the stocking rate, are normally absorbed by body weight loss during the winter or dry periods, and gain during the summer or rainy periods. Thus, cows and ewes managed under extensive grassland systems are exposed to a structural underfeeding system (i.e. annual periods of feed restriction due to low herbage mass production by native pastures). Reproductive function is aligned closely with food supply, which is easily exemplified by the seasonality of sheep to ensure birth during seasons that are favourable for lamb survival. While the initial events of the reproductive process (ovulation/fertilization/early gestation) do not demand relevant amounts of energy, the requirements during late gestation and lactation are considerable, and disruptive events may be life- threatening to the mother and the offspring. However, since the initial reproductive events are sensitive to fluctuations of nutrient availability, the efficient use of energy of the adult female in the reproductive cycle is maximized. Although meat production has increased during the last decades (FAOSTATS, 2017), reproductive efficiency remains low, with calving rates around 60 to 65 % (Pallarés *et al*., 2005). In beef cows, poor nutritional status (reflected in low body condition scores, BCS) together with calf presence/suckling, determines long postpartum anestrus, early embryonic death, reduced pregnancy and weaning rates (Stagg *et al*., 1998; Hess *et al*., 2005; Diskin and Morris, 2008).

In mixed rain-fed dairy systems in which herbage is the primary diet component, in contrast with indoor systems (total mixed ration, TMR), prediction of nutrient availability is complex since it includes uncertainties associated with grazing. Moreover, grazing dairy cows do not take in sufficient dry matter intake (DMI) to sustain the high milk production that could be achieved with their actual genetic potential (Kolver and Muller, 1998). Maximizing DMI is crucial to achieve the desired milk production as well as to minimize the magnitude and duration of the negative energy balance (NEB). The NEB is known to decouple energy requirements of lactation and nutrient supply that takes place during the transition period (Drackley, 1999). The biological processes affected after decades of selection for milk production resulted in physiological undernutrition during early lactation (Chilliard, 1999). Although several reports have showed the abrupt decline in fertility in dairy cows worldwide (Butler, 2000; Lucy, 2001), the improved management in nutrition, health and reproduction, and the genetic selection for health and fertility traits, have changed this trend in the last years in some countries (Berry *et al*., 2014; Thatcher, 2017).

The present review will focus on the effects of NEB on reproductive performance of adult female ruminants, with emphasis on both grassland and mixed rain-fed grazing farming systems. Whilst early investigations focused on the effects of nutrition on the hypothalamic-pituitary axis, studies of the last decade have tested the hypothesis that nutritional signals (metabolic hormones) also exert direct effects at peripheral levels. The potential causes of reproductive failure include impaired folliculogenesis, abnormalities of the ovum/embryo, luteal inadequacy and/or failure of the supply of progesterone to the uterus, abnormal functionality of the reproductive tract and disruption of maternal recognition of pregnancy.

## Ruminant metabolic adaptation to undernutrition: endocrine signals

**Figure 1:**
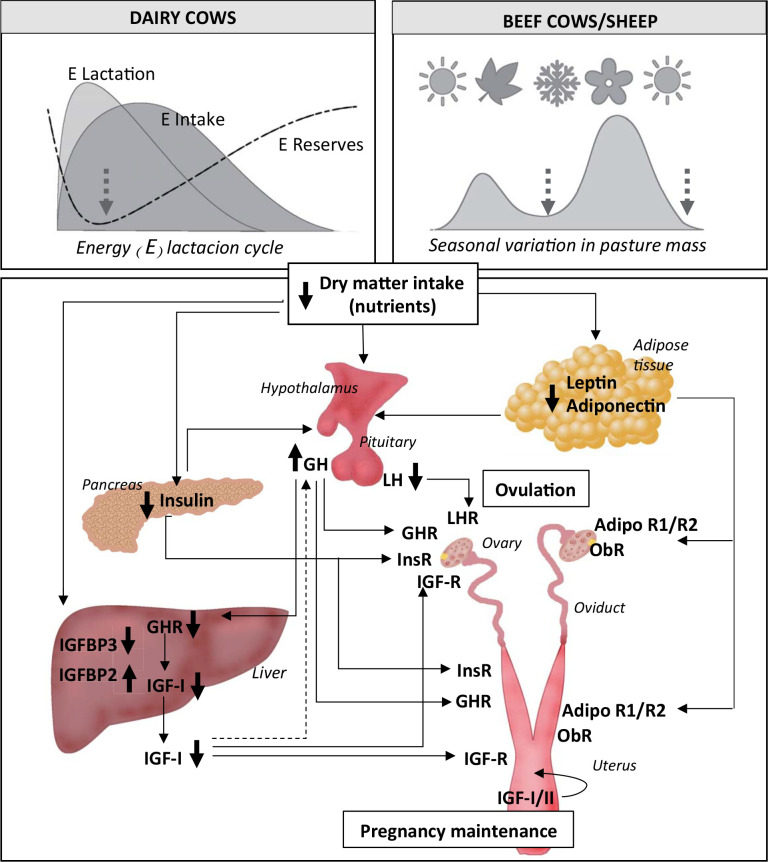
Simplified model of metabolic influences on reproduction of the female adult ruminant. While undernutrition in grazing beef cows and ewes is the result of energy intake below their requirements associated with the seasonal variation in pasture mass, in grazing dairy cows the negative energy balance (NEB) is the consequence of the increased demands for lactation and the low dry matter intake exacerbated by the need to get sufficient intake from pasture and the extra grazing energy costs. Grazing management interacts with parity in the metabolic adaptation to restricted dry matter intake or NEB that also depends on body reserves (metabolic memory). Reproductive events are affected by how and when this adaptation takes place. The nutrient flux affects all tissues and nutrient partitioning is modulated by the endocrine signals. Peripheral tissues (liver, adipose tissue, pancreas and others) secrete signals that not only regulate metabolic pathways, but also inform of the energy status. Hormones interact with each other by regulating their synthesis/secretion to ensure a coordinated regulation of energy partitioning. A complex system in the hypothalamus senses nutrient restriction and regulates voluntary feed intake, nutrient partitioning and reproductive function accordingly. The reproductive axis will respond both hierarchically by regulation of hypothalamus-pituitary gland and at follicular level (i.e., hormone receptors of the metabolic hormones) sensing the metabolic status to overcome the first reproductive limitation in the adult female, which is the pospartum/seasonal anestrus. The endocrine signals have also direct effects on the reproductive tract where they may tip the balance towards pregnancy success or failure. Broken lines: decrease, i.e., negative feedback IGF-I/GH, which is diminished and contributes to higher GH concentrations. Solid lines: increase (positive effect).

**Figure 2:**
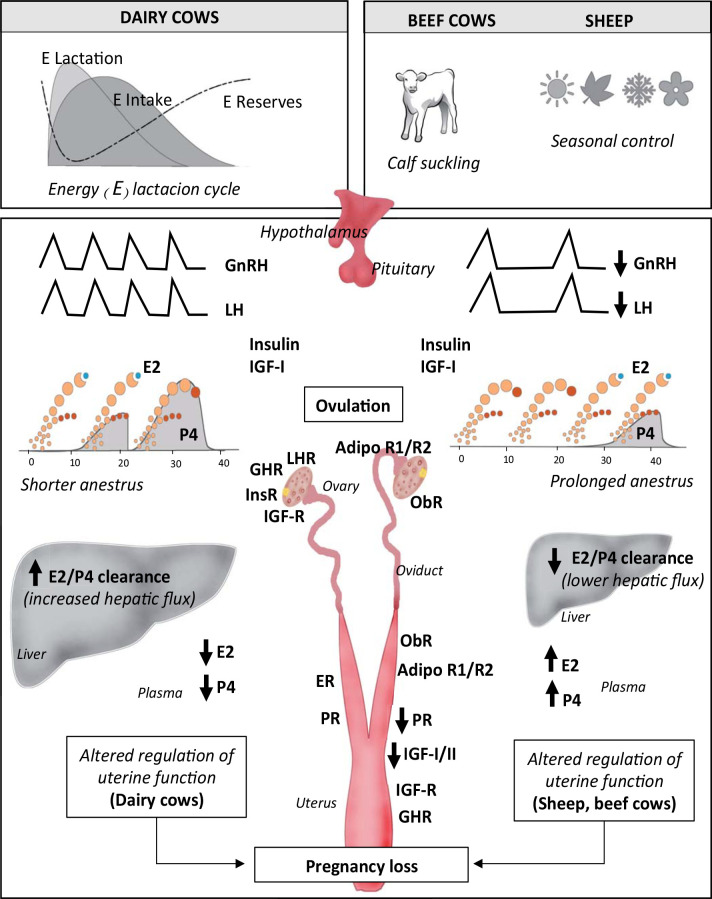
Proposed model of reproductive responses to negative energy balance (NEB) and environment in dairy and beef cows and ewes. Although dairy cows usually present a more profound NEB due to lactation requirements than beef cows and ewes grazing pastures, the later may suffer a more prolonged restricted nutrient availability. Beef cows and ewes present low frequency of LH pulses (suckling or seasonal inhibition, respectively) and undernutrition prolongs pospartum or seasonal anestrus, respectively. Although severe NEB also prolongs pospartum anestrus in dairy cows, is comparatively shorter. Body reserves affect the length of pospartum (dairy and beef cows) and seasonal anestrus (ewes). Once ovulation has occurred, the sequential preparation of the oviducts and uterus by E2 and P4 sustains embryo growth. Clearance of E2 and P4 is affected by liver metabolic flux: in dairy cows the high hepatic flux (intake-lactation) is related to low steroid plasmatic concentrations, whereas in ewes undernutrition is associated with high steroid circulating concentrations (no data as such was found in beef cows). In both dairy cows and sheep, data indicate that uterine sensitivity to P4 (PR) is diminished in NEB, as well as uterine sensitivity to other metabolic hormones. These findings may explain the embryo losses. Embryo mortality seems to be more important in the high producing dairy cow than in beef cows and ewes. The schematic representation of follicular waves in dairy and beef cows has been taken from Crowe *et al*. (2014).

The metabolic adaptation to undernutrition implies adjustments in nutrient partitioning that are regulated by complex signalling pathways among organs and tissues, and depends on multiple intrinsic and extrinsic factors (see Fig. 1). Faced with nutritional deficiency, and depending on its severity, the natural physiological response is to renounce reproduction. As an immediate response in the adult female, cyclicity would be compromised; if this limitation is overcome, the next challenge is pregnancy success and/or the number of offspring (Scaramuzzi *et al*., 2006). Even among sequential reproductive events, undernutrition influences may tip the balance towards success or failure in certain steps of the reproductive cycle. Although biological processes are similar among sheep and cows, ruminant females may show different adaptive mechanisms depending on the species and their environments. For instance, the reproductive responses of the adult female to NEB include the increase in the duration of seasonal anestrus (ewes), postpartum anestrus (cows), decreased fertilization rate and/or increased embryo mortality (Butler, 2000; Hess *et al*., 2005; Forcada and Abecia, 2006; Diskin and Morris, 2008; see Fig. 2). The outcome will vary depending on the energy status and the presence of the offspring (Short *et al*., 1990; Stagg *et al*., 1998). Moreover, the metabolic and reproductive responses to undernutrition depend on recent (feeding level) or more longer term metabolic history (reflected in the body reserves, Chilliard *et al*., 2005). Thus, to understand the influences of undernutrition and metabolism on female reproduction, these aspects have to be considered, as changes in nutrient flux will affect reproductive processes.

Undernutrition and/or NEB in the female ruminant is characterized by decreased blood glucose, insulin and insulin-like growth factor-I (IGF-I), and increased concentrations of non-esterified fatty acids (NEFA; Ciccioli *et al*., 2003; Meikle *et al*., 2004a; Lake *et al*., 2006; Sosa *et al*., 2006a). These metabolic adaptations are coordinated not only by changes in the plasma concentration of key hormones, but by tissue- specific variations in hormonal sensitivity and responsiveness (Bauman, 2000; see Fig. 1). During NEB, the growth hormone (GH)-IGF axis is uncoupled in the liver, resulting in a reduction of circulating IGF-I, despite high GH concentrations (Kobayashi *et al*., 1999). This uncoupling may be the result of a state of hepatic resistance to GH due to decreased mRNA expression of the GH receptor (GHR), especially its isoform 1A, which occurs during NEB in dairy cows (Kobayashi *et al*., 1999). The increased GH concentration in early lactation promotes mobilization of NEFA from adipose tissue and their oxidative use by the rest of the body (Bauman, 2000; Block *et al*., 2001). Thus, anabolism is inhibited directly by the decrease in insulin concentrations and increased insulin resistance of tissues, and indirectly by the lack of insulin stimulation of GHR that limits hepatic synthesis of IGF- I (Butler *et al*., 2003; Rhoads *et al*., 2004; Fig. 1). Information about the effect of undernutrition/NEB on the hepatic molecular mechanism that explains the uncoupling of GH-IGF axis may differ according to species and/or management. Indeed, although blood IGF-I concentrations were decreased during NEB, no reduction in hepatic *GHR-1A* and *IGF-I* mRNA were observed in beef cattle and sheep (Jiang *et al*., 2005; Sosa *et al*., 2006a; Astessiano *et al*., 2014; Laporta *et al*., 2014). In addition, the activity of IGF-I is modulated by complex interactions with specific binding proteins (IGFBPs) that alter the availability of the active growth factor and its cellular receptors (Jones and Clemmons, 1995). Most IGF-I is bound to IGFBP3 and the acid-labile subunit in a ternary complex. The NEB is associated with decreased IGFBP3 and increased IGFBP2 shifting this binding to IGF- I/IGFBP2 complex and reducing the half-life of IGF-I (Jones and Clemmons, 1995; Laporta *et al*., 2014). In our experiments, plasma IGF-I turned out to be the best marker integrating the static (body reserves) and acute effects of nutrition in sheep (Fernández-Foren *et al*., 2011), dairy (Meikle *et al*., 2004a; Adrien *et al*., 2012) and beef cows (Soca *et al*., 2013a; Laporta *et al*., 2014). Indeed, Adrien *et al*. (2012) suggested that insulin profiles were associated more to day-to-day nutritional inputs, while IGF-I profiles more likely reflected the changes in energy balance. Both, insulin and IGF-I, affect the reproductive axis at central (hypothalamus- pituitary gland) and peripheral (gonads, reproductive tract and embryo) levels (Fig. 1).

The adipose tissue plays a role not only in energy storage, but is also an active endocrine tissue sensing metabolic status. Leptin and adiponectin concentrations have been proposed as indexes of metabolic status, as well as metabolic signals to the reproductive system (Blache *et al*., 2000; Kim *et al*., 2011). Plasma concentrations of leptin decrease with undernutrition in sheep and cows (Delavaud *et al*., 2000; Ciccioli *et al*., 2003; Meikle *et al*., 2004a; Sosa *et al*., 2006a; Fig. 1), which facilitates a decrease in metabolic rate and enhances voluntary feed intake (Ingvartsen and Boisclair, 2001). As leptin is mainly synthetized by adipocytes, the decrease in leptin concentrations after restriction in dairy and beef cows and ewes is greater in females with more adipose tissue that also have higher initial concentrations of leptin (Meikle *et al*., 2004a; Fernández-Foren *et al*., 2011; Astessiano *et al*., 2014). Data on adiponectin in undernourished female ruminants are limited. A negative association between adiponectin concentrations and BCS is reported in dairy cows during the dry period (De Koster *et al*., 2017) and during the postpartum period in beef cows (Astessiano *et al*., 2014). Interestingly, in dairy cows, both leptin and adiponectin concentrations decrease around calving, and increase thereafter (Giesy *et al*., 2012; Singh *et al*., 2014; Astessiano *et al*., 2015). Since adiponectin suppresses gluconeogenesis (Zhou *et al*., 2005), its reduction may imply a physiological mechanism to increase glucose supply to the mammary gland for milk production (Saremi *et al*., 2014).

The regulation of these hormones is interdependent (e.g., each hormone affects the synthesis/secretion of the other) to facilitate crosstalk among tissues regulating metabolism (liver, pancreas, adipose tissue, Fig. 1), as reported for these species and rodents (Wabitsch *et al*., 1996; Rhoads *et al*., 2004; Yoshida *et al*., 2007). Thus, the different metabolic responses to undernutrition found among studies can be attributed, among other factors, to differences in body reserves. Indeed, differential BCS responses induced experimentally in sheep (Fernández-Foren *et al*., 2011), beef (Ciccioli *et al*., 2003) and dairy cows (Chagas *et al*., 2006; Adrien *et al*., 2012) were associated with different metabolic responses. Even if underfed ewes had decreased glucose concentrations, insulin concentrations decreased abruptly immediately after undernutrition in lean BCS (<2.25, scale 0-5) ewes, while in moderate BCS (>2.75) ewes the decrease in insulin concentrations was observed two weeks after the start of the treatments (Fernández-Foren *et al*., 2011). Thus, metabolic adaptation to feed restriction depends on body reserves: females with very low energy stores respond rapidly (in terms of metabolites/hormones) to DMI, while the response of animals with greater energy stores seems to be somewhat delayed.

In grazing dairy cows, pre-partum IGF-I and/or leptin concentrations were associated with the level of body reserves in both naturally occurring or induced BCS (Meikle *et al*., 2004a; Chagas *et al*., 2006; Adrien *et al*., 2012). In contrast, subtle or no associations were detected between pre-partum BCS and leptin/IGF-I concentrations after calving in the latter studies. Increased leptin and IGF-I concentrations after calving were reported in response to greater concentrate intake (Reist *et al*., 2003). Indeed, greater plasma insulin and IGF-I concentrations were found in dairy cows fed a TMR compared to grazing dairy cows, even if offered more than 35 kg DM/cow/day of herbage and also supplemented to cover maintenance energy requirements plus 10 liters of milk (Meikle *et al*., 2013). This was consistent with the higher nutrient density of TMR diets and no extra requirements to cover grazing activities or walking. When beef cows were classified according to their BCS at calving (<3.5≥, scale 1-8), IGF-I concentrations during prepartum were greater in moderate than low-BCS cows, with no differences during the postpartum (Astessiano *et al*., 2014). In addition, beef cows grazing high herbage allowance of native pastures during the annual gestation-lactation cycle presented greater BCS and higher IGF-I concentrations than cows grazing low herbage allowance, but also serum IGF-I increased in early spring in response to pasture availability and energy balance only in the high allowance group (Laporta *et al*., 2014). Similarly, when temporary suckling restriction was applied to primiparous beef cows two months after calving, the increase in IGF-I concentrations was greater in cows with moderate (≥4, scale 1-8) *vs*. low (≤3.5) BCS at calving (Soca *et al*., 2013a). Moreover, the endocrine response of these cows to flushing (supplementation for 22 days immediately after suckling restriction) was dependent on BCS at calving, as IGF-I and insulin concentrations increased in moderate BCS cows but did not change in low BCS cows. Overall, the endocrine response to nutritional management depends, at least partially, on body reserves.

In addition, metabolic adaptation to lactation is affected by age (parity): primiparous cows, which have not reached their adult body size and continue growing during pregnancy and lactation, present metabolic differences respect to older cows (Wathes *et al*., 2007). The competing demands of the mammary gland are superimposed on the requirements for growth, and both insulin and IGF-I stimulate growth. The profiles of these hormones and metabolites, such as NEFA, during the transition period according to parity have been inconsistent (Vandehaar *et al*., 1999; Meikle *et al*. 2004a). Wathes *et al*. (2007) modelled metabolic traits, milk yield and BCS, and reported greater IGF-I concentrations in primiparous cows, which may limit nutrient partitioning into milk. In contrast to multiparous cows, there was no relationship between insulin concentration and milk production in primiparous cows. The authors suggested that insulin is less important in controlling the relative partitioning of nutrients between body tissue and milk synthesis, possibly due to the prevailing higher IGF-I concentration in primiparous cows still growing. Moreover, body reserves are usually related to parity, as primiparous dairy and beef cows under pasture-based systems present better BCS than multiparous cows probably for not having the energy demands of a previous lactation. Thus, prepartum leptin concentrations are generally higher in primiparous cows (Meikle *et al*., 2004a; Wathes *et al*., 2007). On the other hand, a steeper decrease in body reserves and leptin concentrations was observed during early lactation in primiparous cows when compared to multiparous cows (Meikle *et al*., 2004a). As both categories are usually managed together under grazing conditions, this may be also explained by a dominance effect for food availability (Grant and Albright, 2001). These confounding factors should be considered since they are at the basis of the poor reproductive performance.

## Follicular and luteal function and physiology of the reproductive tract

Follicular diameter has been associated positively with acquisition of oocyte competence (Arlotto *et al*., 1996) and with enhanced embryo growth and development since the corpus luteum (CL) originated from these follicles secretes more progesterone (P4) concentrations to improve maintenance of pregnancy (Vasconcelos *et al*., 2001; Perry *et al*., 2007). Reduced feed intake has been associated with decreased follicle size in cows (Burns *et al*., 1997). Undernourished ewes presented a reduced number of large follicles compared to control ewes (McNeilly *et al*., 1987). Moreover, undernourished ewes presented larger follicles in the static phase that were functionally altered because there was no inhibition in development of subordinate follicles (Sosa *et al*., 2010).

The ability of the dominant follicle to grow and ovulate depends on LH pulsatility as well as concentration of many growth factors (e.g, IGF family) and nutrients (Fortune *et al*., 2004; Fig. 2). The changes that take place in the dominant follicle (e.g, LH sensitivity and content of steroidogenic enzymes) allow 17-ß estradiol (E2) secretion and subsequent ovulation (Crowe *et al*., 2014). Thus, undernutrition and/or NEB delays ovulation by inhibition of LH frequency and diminished concentrations of IGF-I/insulin and other nutrients, which reduce E2 production by the dominant follicles (Butler, 2000; Fig. 2). Follicular fluid plays a critical role whereby the microenvironment impacts on oocyte development and future embryo quality (Fortune *et al*., 2004; Revelli *et al*., 2009). Increased concentrations of E2 and E2/P4 ratio in follicular fluid have been associated with improved follicular growth and follicle dominance, oocyte quality and pregnancy outcome (Revelli *et al*., 2009). Differences in metabolomic profiles of follicular fluid in cows with different energy balances have been reported (Forde *et al*., 2015) and some components have been associated (positively: alanine and linolenic acid; negatively: total fatty acids and urea) with oocyte competence (Matoba *et al*., 2014).

Beef cows with moderate BCS at calving or grazing high herbage allowance had greater maximum diameter of dominant follicles during the postpartum when compared with low BCS cows or low herbage allowance, respectively (Quintans *et al*., 2010; Carriquiry *et al*., 2011). The E2/P4 ratio did not differ in the preovulatory follicle of beef cows grazing high vs. low herbage allowance. However, intrafollicular IGF-I concentrations were greater in cows grazing high herbage allowance of native pastures consistent with an earlier postpartum ovulation in these cows (Carriquiry *et al*., 2011). Likewise, there is an inverse relationship between plasma IGF-I/insulin and duration of postpartum anestrus in beef and dairy cows (Lucy, 2001; Meikle *et al*., 2004; Quintans *et al*., 2010; Soca *et al*., 2013a; Laporta *et al*., 2014).

The magnitude of NEB and its association with the different types of anestrus have been reviewed before (Peter *et al*., 2009). The first postpartum follicular wave takes place within 10-14 days after calving and the fate of the dominant follicle will depend on LH pulsatilty and the availability of many growth factors within the follicle. Thus, the first pospartum ovulation in well-managed dairy cows will take place within the first month after calving (Crowe *et al*., 2014; Fig. 2). In grazing dairy cows (n = 824 in 7 commercial herds) with none, one or two previous calvings, milk P4 was determined twice weekly for 90 days after calving to monitor luteal activity: 53% of the cows ovulated within 30 days after calving, 14% from 30 to 40 days, and lower percentages were found until 90 days after calving, while 13% of the cows did not ovulate during this period (Meikle, 2018; Facultad de Veterinaria, Udelar, Montevideo, Uruguay; unpublished data). Although dairy cattle usually present a more profound NEB due to lactation than beef cattle grazing pastures, the latter suffer a prolonged restricted nutrient availability in addition to suckling (Fig. 2). The major physiological difference between dairy and beef cows during early postpartum is the lower frequency of LH pulses in beef cows due to suckling inhibition and presence of calf (see Fig. 2). While dairy cows may ovulate in the first follicular wave, in beef cows if nutrition is adequate, the first postpartum ovulation takes place on the third follicular wave (~30 days), but if beef cows present poor body condition, ovulation is delayed (~70-100 days, reviewed by Crowe *et al*., 2014).

The resumption of ovarian cyclicity after calving associated with body reserves is well documented. Indeed, in grazing production systems dairy and beef cows with better BCS during the pre and postpartum periods, or with better BCS at calving, had an earlier first postpartum ovulation (Meikle *et al*., 2004a; Quintans *et al*., 2010; Soca *et al*., 2013b). Soca *et al*. (2013b) determined that the length from calving to first ovulation in beef cows decreased by 49 days for each incremental unit of improvement of BCS at calving (scale 1 to 8). Stagg *et al*. (1995) reported a prolonged period of anestrus in cows with poor body condition. Likewise, increased BCS advanced first ovulation by 59 days in high vs. low herbage allowance cows (Laporta *et al*., 2014). Although less data on postpartum resumption of ovarian cyclicity is available in sheep, it was shown that the first postpartum ovulation in the breeding season was delayed in undernourished ewes with low BCS (<2.75, scale 0 to 5) at lambing in comparison to undernourished ewes with moderate BCS (>2.75) or control ewes (Sosa *et al*., 2006b). The relevance of BCS also was documented in its effect on the length of seasonal anestrus and ovulation rate, as ewes with a moderate BCS (>2.75) had a shorter seasonal anestrus (64 days) than ewes with a low BCS (<2.75; 113 days) and a higher rate of double ovulation as observed by the greater mean number of CLs (1.67 *vs*. 1.08 CL, respectively), especially in the transition between seasonal anestrus and the breeding season (Forcada and Abecia, 2006). These data show that the resumption of postpartum/seasonal ovarian cyclicity depends on female body reserves.

Parity is a relevant factor when considering postpartum resumption of ovarian cyclicity, which occurred 16-23 days later in primiparous than multiparous grazing dairy cows (Meikle *et al*., 2004a; Adrien *et al*., 2012). This is consistent with the lower percentage of inseminated primiparous cows in the first 80 days after calving compared to multiparous cows (39.8 *vs*. 50.4%, 3772 dairy cows from 12 commercial farms; Cruz, 2018; Facultad de Veterinaria, Udelar, Montevideo, Uruguay; unpublished data). On the other hand, conception rate at first service in the later study was higher in primiparous cows (49.4 *vs*. 38.1%). Similar data was reported in pasture-based dairy systems (for reviews, see Rhodes *et al*., 2003 and Roche *et al*., 2011). In high-producing confined dairy herds, Santos *et al*. (2009) reported that multiparous cows were more likely to start ovarian cyclicity earlier than primiparous cows, but conception rate on day 30 after AI (~100 days after calving) was higher in primiparous cows (41.1 *vs*. 36.1%). Thus, although NEB heavily influences postpartum resumption of ovarian cyclicity, it only explains partially the decreased reproductive efficiency due to nutrition.

Once ovulation and fertilization has occurred, an adequate functionality of the oviduct and uterine horn is essential for the maintenance of pregnancy. The morphological, histological and biochemical changes of the reproductive tract are modulated by the fluctuating concentrations of E2 and P4 acting through their specific receptors in the target tissues, which also vary during the estrous cycle and early pregnancy (Meikle *et al*. 2001, 2004b; Robinson *et al*. 2001; Binelli *et al*., 2018). Thus, their function can be modified by manipulating the growth of the preovulatory follicle and its associated E2 secretion, as well as the consequent CL formation and associated P4 production (Mesquita *et al*., 2015; Ramos *et al*., 2015). Indeed, a positive association between preovulatory concentrations of E2 and the duration of proestrus regarding the uterine environment and fertility was found (Bridges *et al*., 2010, 2012). Pugliese *et al*. (2016) showed that the diameter of the preovulatory follicle and its blood flow and size, vascularization of CL and pregnancy rates were greater in beef cows ovulating large follicles. Moreover, Ramos *et al*. (2015) reported decreased uterine redox capacity in cows that ovulated a smaller follicle during early diestrus, and the authors suggested that it might be one of the causes of the reduced fertility found in these animals. Recently, de la Mata *et al*. (2018) demonstrated that extending the proestrus interval increased the rate of the dominant follicle growth from the time of P4 device removal to ovulation, the luteal area and serum P4 concentrations, which were associated with endometrial differences in PR immunostaining, *PR* and *IGF-I* mRNA expression, and pregnancy rates.

Progesterone supports the secretory function of the endometrium that sustains conceptus elongation and further implantation (for a review see Spencer *et al*., 2007). Moreover, there is a positive relationship between P4 concentrations during the early luteal phase and the synthesis of the embryo signal, interferon tau (IFN-τ). The IFN-τ, by altering uterine gene expression, modifies the episodic PGF_2α_ release that is responsible for the luteal regression (Mann and Lamming, 2001). Different authors have managed to modify endometrial function and improve embryo growth by injecting P4 during the early luteal phase in the female ruminant (Robinson *et al*., 1989; Forde *et al*., 2009). However, results are not consistent as effects depend on many factors, e.g., the timing of start and the duration of P4 supplementation affect CL and embryo development in beef heifers (Parr *et al*., 2017). Moreover, Bisinotto *et al*. (2015) reported that P4 supplementation during timed AI programs in cows without a CL at the start of the treatment improved pregnancy per timed AI, but P4 supplementation had no positive effect in cows that had a CL at time of supplementation.

In sheep, P4 concentrations are 800-fold higher in the ovarian than in the jugular vein, but did not differ between high and low feeding levels (reviewed by Abecia *et al*., 2006). On the other hand, plasma P4 concentrations were negatively associated with the level of feed intake, with higher P4 concentrations in underfed ewes likely due to lower liver steroid metabolism, thus a lower clearance rate (Rhind *et al*., 1989b; Lozano *et al*., 1998; see Fig. 2). In dairy cows, increased feed intake has been associated with the increase of E2 and P4 clearance (Sangsritavong *et al*., 2002), which can contribute to inadequate endometrial functionality. On the other hand, plasma P4 circulating concentrations may not reflect actual P4 concentrations in the reproductive tract. Indeed, in the ruminant, P4 is transferred locally from the ovarian/oviductal veins to the uterine arterial segments adjacent to the ovary (Weems *et al*., 1989). Although receptors specifically bind hormones with high specificity, few studies have analysed P4 and its receptor (PR) concentrations where transfer systems are in place in the fed restricted female ruminant. The region and side of the uterus affect transcript abundance in bovine endometrium (Sponchiado *et al*., 2017). Also, a differential oviductal gene expression associated with CL location was reported (de Brun *et al*., 2013) in agreement with greater P4 concentrations in the ipsilateral oviduct of recipient ewes (Graña *et al*., 2018; Facultad de Veterinaria, Udelar, Montevideo, Uruguay; unpublished data). These specific molecular differences according to location are of functional importance: when sheep IVF zygotes were transferred to oviducts ipsilateral and contralateral to the CL on day 1, a greater recovery rate and a lower proportion of degenerated embryos were found in the ipsilateral uterine horn on day 6 (de Brun *et al*., 2016a).

In cyclic undernourished ewes, higher plasma but lower endometrial P4 concentrations were found on day 5 (Lozano *et al*., 1998). This was later explained by the lower expression of *PR* mRNA, and lower PR protein abundance and binding capacity in uterus/oviduct of undernourished ewes (Sosa *et al*., 2006a; Fig. 2). As P4 regulates endometrial gene expression, differences in uterine P4 sensitivity (PR) were associated with different functionality and early embryo growth, as reported for different sheep breeds (Sequeira *et al*., 2016). Indeed, undernutrition in sheep was also associated with a lower expression of *IGF-I* mRNA in uterus and *IGF-II* mRNA in oviduct at day 5 of pregnancy (de Brun *et al*., 2013; Fig. 2). Overall, these findings support the concept that local P4 concentrations (i.e. oviducts and uterine horns), and the consequent differential regulation of the reproductive tract physiology, may explain the higher embryo losses found in undernourished ewes (Rhind *et al*., 1989a, b).

The effect of underfeeding and/or NEB on gene expression in the reproductive tract of cyclic and/or postpartum cows has also been investigated. Endometrial and oviductal gene expression of IGF signaling pathways and several metalloproteinases were altered in dairy cows in severe NEB two weeks after calving (Wathes *et al*., 2011). It was suggested that altered postpartum functionality of the tract might affect tissue repair with consequent lower fertility. Less data are available regarding NEB on uterine gene expression in lactating cows at breeding time. Rhoads *et al*. (2008) reported no changes in mRNA endometrial expression of *GHR* and *IGFBP2* in lactating cows on 40, 80, 120 and 160 days after calving, although higher *IGF-I* mRNA levels were found on day 160; it was suggested that days in milk had a small effect on uterine gene expression. In a recent study in lactating dairy cows, Astessiano *et al*. (2017) reported greater intercaruncular endometrial mRNA expression of *IGF-I, IGFBP3, PR* and adiponectin receptors on day 7 of the estrous cycle at the end of the voluntary waiting period when cows fed a TMR and high herbage allowance were compared to medium and/or low herbage allowance cows (Fig. 2). Thus, the ruminant endometrium seems to be able to sense the metabolic status and to adapt its physiology accordingly, thereby determining possible success or failure of pregnancy.

## Uterine functionality during early pregnancy

Although the preimplantation embryo is to a certain extent metabolically autonomous, the fate of the embryo is affected by the nutritional status of the maternal unit. Maternal undernutrition in sheep results in retarded embryonic development at 8 to 11 days after mating and reduced pregnancy rates after 2 weeks of pregnancy (Rhind *et al*., 1989a; Abecia *et al*., 2006). Although relatively high fertilization rates are found in dairy cows, conception rates are low (Lucy, 2001; Diskin and Morris, 2008). The proportion of embryos recovered from lactating cows was lower than from dry cows, consistent with the lower embryo quality reported for high-producing dairy cows (Sartori *et al*., 2010). Data suggest an effect of the energy balance on the functionality of the reproductive tract as reviewed previously (Sartori *et al*., 2010; Lonergan *et al*., 2016).

Much focus has been put on the effects of NEB around the moment of maternal recognition of pregnancy (i.e., day 14 in sheep, and day 17 in cows). In dairy cows, at day 17-intercaruncular endometrium, genes were differentially expressed according to pregnancy and lactation (Cerri *et al*., 2012; Thompson *et al*., 2012). Lactation altered the metabolic status (lower glucose and IGF-I plasma concentrations) and decreased P4 plasma concentrations (Thompson *et al*., 2012). The presence of the embryo had profound effects on endometrial expression of genes involved in immune pathways, but lactation also upregulated genes related to immunoglobulins, so that it was suggested that lactation could cause an immune imbalance with potential negative effects on conceptus survival (Cerri *et al*., 2012). Moreover, lactation affected the expression of genes involved in glucose homeostasis suggesting that it could be deleterious for the embryo. Lesage-Padilla *et al*. (2017) demonstrated that day 19-intercaruncular endometrium of pregnant lactating cows presented greater mRNA expression of oxidative stress-related genes when compared to pregnant dry cows, suggesting that lactation is associated with an increase in reactive oxygen species in the endometrium. As endometrial expression of conceptus-regulated genes was not affected by the metabolic status, it was suggested that the endometrial ability to respond to embryonic signals when implantation occurs seems not to be affected by maternal metabolism (Lesage-Padilla *et al*., 2017). No reports on the effect of NEB on local P4 endometrial concentrations and its association with uterine gene expression in pregnant or cyclic dairy cows have been found.

Lower fertilization (27%) and transferability (30%) rates were observed in undernourished superovulated donor ewes when compared to well-fed ewes (Abecia *et al*., 2015). When only good quality embryos (n = 2) from undernourished and control superovulated donor ewes were transferred to undernourished and control recipient ewes, no differences in pregnancy rates were found at day 18, but recipient undernourished ewes had a higher occurrence of late embryonic mortality (from days 18-40; de Brun *et al*., 2016b). These ewes had lower plasma insulin and P4 concentrations during the early luteal phase, which may have affected conceptus development leading to pregnancy loss after day 18. Overall, data suggest that when morphologically good quality embryos are transferred, maintenance of pregnancy relies on the nutrition of the mother regardless of embryo origin.

Embryos collected on day 15 of pregnancy from underfed ewes (0.5 maintenance requirements) secreted lower amounts of IFNτ in vitro, and the endometrial tissue collected from those ewes secreted higher amounts of PGF2α than control ewes (Abecia *et al*., 2006). Since this was accompanied by a reduction in embryo survival, it was suggested that the lower fertility observed in underfed ewes could be mediated through altered signals of maternal recognition of pregnancy. Further studies showed no effects of undernutrition on the intercaruncular endometrial expression of candidate genes involved in mechanisms of maternal recognition of pregnancy in pregnant ewes (such as PR, estrogen and oxytocin receptors, cyclooxygenase 2 and members of the IGF family) on day 14 (Sosa *et al*., 2009). It was suggested that conceptuses present in the uterus of undernourished mothers managed to elicit effects similar to those in well-fed pregnant ewes. In order to gain in-depth knowledge of the underlying mechanisms, we have studied the uterine transcriptome of the same ewes (de Brun *et al*., 2016c). Interestingly, pregnancy in control ewes upregulated the gene expression of the citrate cycle and oxidative phosphorylation pathways, suggesting increased energy use by the endometrium to maintain pregnancy, as was previously reported in pregnant dairy cows (Cerri *et al*., 2012). In contrast, pregnancy in undernourished ewes did not affect these pathways, but upregulation of catabolic pathways for fatty acids and amino acids was observed (de Brun *et al*., 2016c). Moreover, undernutrition in pregnant ewes induced a 9-fold change in acyl-CoA synthetase short- chain family member 2 (an enzyme that converts acetate to acetyl-CoA) mRNA expression compared to control pregnant ewes. This differential response is indicative that endometrial cells utilize energy from these pathways, probably sparing glucose for utilization by the embryo. The immune system is also critical for proper embryo development and is activated in pregnant animals from both nutritional groups. Intercaruncular endometrium from pregnant ewes had a 3.4-fold or 2.0- fold greater expression of interferon-induced helicase C domain1 (*IFIH1*) than cyclic ewes in control or undernourished ewes respectively (de Brun, 2018; Facultad de Veterinaria, Udelar, Montevideo, Uruguay; unpublished data). The IFIH1 gene modulates local immune cells in the endometrium during pregnancy (Song *et al*., 2007). In the same study, the chemokine CXCL10, a promoter of trophoblast growth migration and adhesion (Nagaoka *et al*., 2003) was upregulated more than 6 and 3-fold in pregnant vs. cyclic control and undernourished ewes respectively (de Brun, 2018; Facultad de Veterinaria, Udelar, Montevideo, Uruguay; unpublished data), which could be associated with less efficient growth or adhesion of the embryo in undernourished ewes. Genes participating in the biosynthesis of unsaturated fatty acids were highly downregulated in undernourished pregnant compared with control pregnant ewes, which could be consistent with an energy-sparing mechanism. On the other hand, as unsaturated fatty acids are precursors for various eicosanoids and prostaglandins involved in the adhesion of the ovine trophoblast to the endometrium and in vascular permeability (Salleh, 2014), the downregulation observed in pregnant underfed ewes may be related with the greater late embryo mortality detected in undernourished animals (de Brun *et al*., 2016b). Overall, the endometrial machinery appears to have an adaptive ability to respond to adverse changes in metabolic status due to feed restriction that is dependent on presence of the embryo.

## Concluding remarks

The ruminant adult female reproductive physiology is highly influenced by the environment and energy intake is a pivotal factor. Depending on the degree of energy deficit, the reproductive adaptation to metabolic distress may range from an increased duration of postpartum or seasonal anestrus to impairment of pregnancy establishment. The metabolic adaptation to NEB depends on body reserves that also modulate reproductive responses. The challenge to improve sustainable livestock production resides in maintaining reproductive efficiency of the female ruminant while facing periods of undernutrition, both in grassland (beef and sheep) or mixed rain-fed farming systems (dairy cows). The goals are to identify the biological reproductive processes that are most affected by NEB, and to determine when and how they can be improved by management complementary to cultural, economic and environmental sustainable systems.
